# BundlED Up: A Narrative Review of Antimicrobial Stewardship Initiatives and Bundles in the Emergency Department

**DOI:** 10.3390/pharmacy7040145

**Published:** 2019-11-01

**Authors:** Maressa Santarossa, Emily N. Kilber, Eric Wenzler, Fritzie S. Albarillo, Ethan J. Sterk

**Affiliations:** 1 Department of Medicine, Division of Infectious Disease, Loyola University Medical Center, Maywood, IL 60153, USA; frialbarillo@lumc.edu; 2 Department of Pharmacy, Maricopa Medical Center, Phoenix, AZ 85008, USA; ekilber@email.arizona.edu; 3 College of Pharmacy, University of Illinois at Chicago, Chicago, IL 60612, USA; wenzler@uic.edu; 4 Department of Emergency Medicine, Loyola University Medical Center, Maywood, IL 60153, USA; esterk@lumc.edu

**Keywords:** antimicrobial stewardship, bundle, emergency department, infectious diseases

## Abstract

Antimicrobial stewardship (ASP) is becoming an increasingly high priority worldwide, yet the emergency department (ED) is an area where stewardship is often neglected. Implementing care bundles, guidelines, and protocols appears to be a rational strategy for ED stewardship given the inherently dynamic and hectic environment of care. Multiple questions still exist such as whether to target certain disease states, optimal implementation of ASP interventions in the ED, and the benefit of unique ED-specific guidelines and protocols. A narrative review was performed on interventions, guidelines, and bundles implemented in the ED setting, in an effort to improve ASP or management of infectious diseases. This review is meant to serve as a framework for the reader to implement these practices at their own institution. We examined various studies related to ASP interventions or care bundles in the ED which included: CNS infections (one study), skin and soft-tissue infections (one study), respiratory infections (four studies), urinary tract infections and sexually transmitted infections (eight studies), sepsis (two studies), culture follow-up programs (four studies), and stewardship in general or multiple infection types (five studies). The interventions in this review were diverse, yet the majority showed a benefit in clinical outcomes or a decrease in antimicrobial use. Care bundles, guidelines, and antimicrobial stewardship interventions can streamline care and improve the management of common infectious diseases seen in the ED.

## 1. Introduction

Antimicrobial stewardship is becoming an increasingly high priority worldwide, due to mounting threats of antimicrobial resistance, a dwindling armamentarium of antimicrobials, and progressively complex patient populations [1]. Antimicrobial stewardship programs (ASPs) are becoming more common, and with the advent of the Joint Commission standard requiring each hospital to possess a formal stewardship program, more data about the benefits of stewardship on patient care and antimicrobial resistance are becoming evident [2]. Although the specific undertakings of ASPs are at the discretion of each individual institution, the CDC recommends implementing policies and interventions to improve antibiotic use, tracking and reporting antibiotic use and outcomes, and education as part of their Core Elements of Hospital Antibiotic Stewardship Programs [3].

An area of intervention that is often neglected by many ASPs is the emergency department (ED), as evidenced, in part, by the estimated 142,000 annual ED visits due to adverse events associated with antimicrobials and the more than 10% of short-term readmissions due to worsening infection [4,5]. The ED is unique from a stewardship perspective, in that it encompasses a diverse patient population with disease states ranging in severity which require a rapid diagnosis and care in a fast-paced clinical environment [6]. The ED also serves as a bridge between the inpatient and outpatient settings, where up to half of all medical care in the United States occurs [7]. Furthermore, clinicians in the ED are often responsible for initiating empiric antimicrobial therapy, often with limited patient-specific clinical information, which lays the foundation for their subsequent admission. This offers a large number of opportunities for stewardship interventions, however, the strategies utilized need to be unique in relation to conventional inpatient antimicrobial stewardship initiatives. May et al. described numerous approaches to antimicrobial stewardship in the ED in an expert commentary [8]. These approaches included education, clinical pathways and guidelines, ED pharmacist support, clinical decision support systems, antimicrobial order forms, post-prescription review, rapid diagnostic testing, shortening duration of therapy, dose optimization, streamlining or de-escalation of therapy, and ED antibiogram development. 

Implementing care bundles, guidelines, and protocols appears to be a rational strategy for ED stewardship given the inherently dynamic and hectic environment of care. Given that the CDC Core Elements of Outpatient Antibiotic Stewardship include the ED and emergency medicine clinicians, many institutions will be initiating or expanding their ASPs interventions to include the ED [3]. Multiple questions still exist such as whether to target certain disease states, optimal implementation of ASP interventions in the ED, and the benefit of unique ED-specific guidelines and protocols. This review aims to address these questions, and to also serve as a framework for those who wish to expand or implement an ED ASP program. 

## 2. Methods

### 2.1. Ethics

This article is based on previously conducted studies and does not involve any new studies of human or animal subjects performed by any of the authors. 

### 2.2. Search Strategy

The aim of this review was to evaluate studies that involved implementing an intervention or bundle to improve antimicrobial stewardship in the ED. This included any type of intervention or infection. We limited our search to focus on studies in adult patients utilizing PubMed via the following search terms: "antimicrobial stewardship AND emergency department", "infection AND bundle AND emergency department", "antimicrobial stewardship bundle AND emergency department", "antimicrobial stewardship intervention AND emergency department", "infection bundle AND emergency department". This process was replicated by two separate authors and the references of included studies were reviewed for completeness. As a result of our search, 25 studies were examined (Table 1). The ASP interventions in the identified studies involved the following: central nervous system infections (one), skin and soft-tissue infections (one), respiratory infections (four), genitourinary tract infections (eight), sepsis (two), culture follow-up programs (four), and stewardship in general or multiple infection types (five). 

## 3. Results

### 3.1. Central Nervous System Infections

Viale et al. conducted a quasi-experimental study which involved implementing a meningitis bundle to patients with suspected meningitis in Italy [9]. The investigators administered supportive care, dexamethasone, a third-generation cephalosporin, and levofloxacin if the patient’s CSF was turbid to all patients with suspected bacterial meningitis in the ED. Emphasis was made to administer the dexamethasone and third-generation cephalosporin within 1 hour of evaluating the patient, even if this occurred before the LP. Eighty-five patients were managed using the new bundle and compared to 92 historical control patients. This intervention led to a numerical reduction in neurologic sequelae (13.9% versus 18.9%, *p* = 0.4) which translated in part into a significant reduction in in-hospital mortality (4.7% versus 14.1%, *p* = 0.04). 

### 3.2. Skin and Soft-Tissue Infections (SSTI)

May et al. conducted a randomized-controlled trial involving implementation of rapid molecular testing for *Staphylococcus aureus* (Xpert MRSA/MSSA SSTI, Cepheid, Sunnyvale CA) following incision and drainage (I&D) of cutaneous abscesses [10]. This study took place in two academic emergency departments. There were 126 patients randomized to the rapid-test group, and 126 patients in the control group. Patients in the intervention group with methicillin-susceptible *S. aureus* received β-lactams more often than controls, and patients in the intervention group who were positive for methicillin-resistant *S. aureus* were more often prescribed anti-methicillin-resistant *S. aureus* antibiotics (93.8% versus 72.2%, absolute difference, 21.5% (95% CI, 10.1% to 33.0%). No difference in clinical outcomes or overall antibiotic utilization was observed. The rapid molecular test had a mean turnaround time of 82 minutes, however, the time to initiate appropriate antibiotic therapy was not examined, and this test did not affect overall antibiotic use. Unfortunately, the Infectious Diseases Society of America (IDSA) Guidelines for the treatment of SSTI were updated after the conclusion of this study, and indicated that routine antibiotic therapy is not recommended for mild SSTIs after I&D. The authors do identify this as a limitation to the study, yet acknowledge the updated guidelines still recommend treatment for certain instances of purulent SSTIs after I&D.

### 3.3. Respiratory Infections

Studies encompassing respiratory tract infections were found to include community-acquired pneumonia (CAP), acute respiratory tract infections, and group-A streptococcal (GAS) pharyngitis. Hortmann et al. described a CAP bundle in a German hospital which involved provider education, checklists, and feedback [11]. This bundle consisted of an extensive education program including both diagnostics and treatment of CAP, and provided handouts to summarize the educational efforts which were published online and in the ED. Providers and nurses were also given monthly feedback on certain performance measures. The bundle resulted in decreased in-hospital mortality (11.3% versus 14.3%, *p* = 0.02), decreased time to the initiation of antimicrobial therapy (72.8% versus 82.7% within 4 hours, *p* = 0.0001), and decreased length of stay (nine versus eight days, *p* = 0.02). In this study, appropriateness of antibiotic therapy was defined according to the German Guidelines for the Management of Lower Respiratory Tract Infections and CAP, since this study took place in Germany. Ostrowsky et al. also implemented a CAP bundle in two EDs, however, this bundle consisted of a treatment algorithm, a "CAP kit" which includes appropriate antibiotics and doses, and preloading an automated dispensing system with antibiotics in the ED [12]. This intervention increased appropriate antibiotic selection in both a pilot ED (54.9% versus 93.4%, *p* = 0.001) and second ED (64.6% versus 91.3%, *p* = 0.004). This intervention, however, did not affect antibiotic administration within 6 hours. Metlay et al. implemented a bundle for acute respiratory tract infections among 16 EDs in a cluster randomized trial, including Veterans Affairs (VA) and non-VA hospitals [13]. The intervention involved performance feedback, provider education, and patient educational materials, including a kiosk in the waiting room and posters or brochures. This resulted in a decrease in antibiotic prescriptions for upper respiratory tract infections and acute bronchitis between intervention sites from 52% in year one to 42% in year two (−10% change, 95% CI, −18% to −2%) but no difference in antibiotic prescriptions between control sites (47% in year one and year two, +0.5% change, 95% CI, −3% to 5%). Overall, there was no change in return visits or patient satisfaction scores as a result of this intervention. Lastly, a quasi-experimental study by Dumkow et al. was conducted at a community teaching ED focusing on GAS pharyngitis [14]. The intervention focused on symptom assessment upon culture follow-up with a focus on antibiotic avoidance for GAS pharyngitis if patients were asymptomatic. This was recommended per IDSA guidelines because patients could be asymptomatic carriers for GAS with a concomitant viral infection. This intervention resulted in a decrease of antibiotic prescribing (97% to 71.3%, *p* < 0.001) and an increase in appropriateness of therapy at follow-up (6.0% to 81.5%, *p* < 0.001). In this study, appropriateness of therapy was defined as symptom assessment at a follow-up visit and the prescribing of β-lactam antibiotics only in the event of symptoms for a guideline-recommended duration. In addition, this intervention did not result in an increase in ED return visits within 72 hours (1.0% versus 5.6%, *p* = 0.121). 

### 3.4. Urinary Tract Infections

Hecker et al. conducted a quasi-experimental study in an academic urban ED to measure an intervention of an electronic urinary tract infection (UTI) order set followed by a prospective audit and feedback intervention [15]. The prospective audit and feedback involved case review by an infectious disease (ID) pharmacist and physician, and direct feedback to ED providers via staff electronic messages. Adherence to guidelines was measured on two-hundred patient visits during three time periods, i.e., prior to stewardship intervention, after the electronic order set implementation, and after the prospective audit and feedback intervention. Overall, this intervention increased adherence to local ED guidelines (44% to 68% to 82%, *p* ≤ 0.015), decreased fluoroquinolone prescriptions (44% to 14% to 13%, *p* < 0.001), and decreased unnecessary antibiotic days of therapy (250 days to 119 days to 52 days, *p* < 0.001). Interestingly, a financial incentive was also offered after implementation of the electronic order set, yet this did not result in a significant increase in adherence to recommended treatments (67% versus 69%, *p* = 0.88). The financial incentive was awarded based on compliance with certain quality metrics, one being use of the UTI order set, however, this was a pre-existing policy and not part of the stewardship initiative in this study. No increases in treatment failures or adverse events were observed as a result of the intervention. Zhang et al. focused on implementation of a pharmacist-led review of all urine cultures with recommendations made to the provider if the patient was non-pregnant and was deemed to have asymptomatic bacteriuria [16]. Of 457 encounters reviewed, 136 patients were found to meet criteria for asymptomatic bacteriuria. Interventions were made for 65% of patients who were discharged with antibiotics, and this resulted in 122/426 (29%) potential antibiotic days saved, resulting from both recommendations to discontinue therapy or to change a prescription. The acceptance rate of pharmacist recommendations to mid-level providers was 93.3%. However, clinical outcomes were not analyzed in this study. Hudepohl et al. conducted a quasi-experimental study involving education on resistance data and preferred antimicrobial therapy via an email to all ED providers and a document posted in the ED [17]. The document focused on prescribing nitrofurantoin for uncomplicated UTIs. This decreased the number of trimethoprim-sulfamethoxazole (13% to 7%, *p* = 0.01) and ciprofloxacin prescriptions (39% versus 26%, *p* < 0.001), as well as decreased the number of ineffective prescriptions, although this was not statistically significant (OR 0.51, 95% CI, 0.17 to 1.52). Percival et al. also conducted a quasi-experimental study focusing on uncomplicated UTIs [18]. The investigators created an ED specific antibiogram and developed an institution-specific treatment guideline. This guideline was disseminated to providers via email, and by direct education to providers by the ED pharmacist. The guideline emphasized the use of nitrofurantoin or cephalexin as first-line options for cystitis. The intervention resulted in an increase in guideline adherent recommendations (44.8% versus 83%, *p* < 0.001), increased nitrofurantoin use (12% versus 80%, *p* < 0.001), and increased effective empiric treatment regimens (74% versus 89%, *p* = 0.05). Similarly, Landry et al. developed a treatment algorithm for uncomplicated UTIs coupled with physician education, which involved direct education to the ED providers and nursing managers [19]. Posters of the algorithm were also displayed in the ED and posted on the intranet. This resulted in an increase in adherence to best practices (41% versus 66% *p* < 0.001) and a change in antibiotic selection which was driven by a decrease in ciprofloxacin utilization. Jorgensen et al. developed a UTI treatment algorithm which emphasized nitrofurantoin as a first line option for uncomplicated UTIs, and offered feedback by ASP to providers [20]. This caused an increase in nitrofurantoin prescriptions (16% to 43%, *p* < 0.001), with a compensatory decrease in cephalexin prescriptions (45% to 10%, *p* < 0.001). Regarding sexually transmitted infections, Rivard et al. implemented the use of a rapid molecular test for the diagnosis of *C. trachomatis* and *N. gonorrhoeae* infections [21]. This diagnostic test resulted in a faster result, and therefore an increase in treatment appropriateness from 60% to 72.5% (*p* = 0.008). This resulted in a predicted cost-savings of approximately $37,000 per year via decreased testing costs, decreased antimicrobial use, and decreased readmissions.

### 3.5. Sepsis

Kalich et al. implemented an antibiotic-specific sepsis bundle with dosing recommendations, education to providers, and antibiotics stocked in an automated dispensing cabinet [22]. This resulted in an increase in the appropriate initial antibiotic choice from 33.9% to 54.8% (*p* = 0.03), however, it did not result in a significant difference in appropriate initial antibiotic choice within one hour (22.6% versus 14.5%, *p* = 0.36). In this study, appropriate antimicrobial therapy options were agreed upon by the investigators according to national guidelines for suspected source of infection and aggregated on a list prior to initiation of the study that blinded investigators would use for reference. Additionally, in order to be considered appropriate, the most broad-spectrum antibiotic must have been initiated first. Viale et al. created an ID consultant sepsis team which was expected to evaluate patients at the bedside within one hour for therapeutic and diagnostic recommendations [23]. This increased the Surviving Sepsis Campaign bundle compliance from 4.6% to 32% (*p* < 0.001), and the appropriateness of initial antibiotic therapy from 30% to 79% (*p* < 0.001). The multivariate regression revealed that being managed by the sepsis team was a protective factor for 14-day all-cause mortality (HR 0.64, 95% CI, 0.43 to 0.94). Interestingly, an increase in the average cost of hospitalization did occur, with an average of 5120 € per patient during the pre-intervention phase and an average of 6745 € per patient in the post-intervention phase. 

### 3.6. Culture Follow-up Programs

Santiago et al. sought to compare culture follow-up by emergency medicine pharmacist review versus nurse in charge review [24]. The median time to initial review was 3 hours (1.0–6.3 hours) in the pharmacist group versus 2 hours (0.3–5.5) in the nurse group (*p* = 0.35). The number of indicated interventions that were not completed in the pharmacist group was 4% versus 47% in the nurse group while the number of patients returning to the ED was not significantly different between the pharmacist and nurse groups. Dumkow et al. implemented a multidisciplinary culture follow-up program involving both pharmacists and physicians [25]. Patients in the culture follow-up group who were uninsured had fewer ED visits within 72 hours as compared with the control group (15.3% versus 2.4%, *p* = 0.044). Overall ED re-visits within 72 hours trended toward a decrease in the intervention group, however, this was not statistically significant. Overall, therapy was modified in 25.5% of evaluated patients. Baker et al. sought to implement a pharmacist managed antimicrobial stewardship program including provider education and culture follow-up [26]. The pharmacist intervention decreased the median time to culture review from three days to two days (*p* = 0.0001), as well as the median time to patient and provider notification from three days to two days (*p* = 0.01). This intervention, however, did not affect overall appropriateness of antimicrobial therapy. Randolph et al. also implemented a pharmacist run culture follow-up program and compared it to the standard which was physician managed culture follow-up [27]. This intervention increased antimicrobials to be modified 15% of the time as compared with 12% pre-intervention. The investigators also measured ED readmissions within 96 hours, and this decreased from 19% pre-intervention to 7% post-intervention (*p* < 0.001). 

### 3.7. Overall Stewardship

Dinh et al. conducted a quasi-experimental study after implementing an ED antimicrobial stewardship program, which included a 0.2 full-time equivalent (FTE) infectious disease physician [28]. This decreased the number of antimicrobials prescribed at consultations from 769 to 580 (*p* < 0.0001). This also increased the guideline compliance from 37% to 53.3% (*p* < 0.00001). Kaufman et al. implemented a front-line intervention involving ED physicians, which was facilitated by the hospital ASP [29]. This resulted in a reduction in antimicrobial use of azithromycin, ceftriaxone, ciprofloxacin, and moxifloxacin. This also reduced the rate of urine cultures by 2.26 cultures per 100 ED visits (*p* < 0.001). Davis et al. implemented a pharmacist-driven antimicrobial optimization service, which was compared to standard nurse-managed cultures [30]. This increased the number of interventions made from 50% to 80% (*p* = 0.01), and also decreased the time to intervention from a mean of 3.4 days to 1.9 days (*p* = 0.81). Borde et al. conducted a quasi-experimental study after the implementation of guidelines and focused discussion groups [31]. The goal of this intervention was to reduce utilization of a third-generation cephalosporin and fluoroquinolones, while encouraging use of penicillin. This intervention decreased third-generation cephalosporin use by an intervention-related decrease of 15.2 recommended daily doses per 100 patient days (RDD/100) (95% CI, −24.08 to −6.311), with a compensatory increase in aminopenicillin/β-lactamase inhibitor use by 6.6 RDD/100 (95% CI, 4.169 to 9.069). Fagan et al. removed ciprofloxacin from the ED antibiotic formulary and instead introduced a suggestion list for antibiotic use in conjunction with point-of-care urine dipstick testing [32]. This intervention significantly decreased ciprofloxacin prescriptions from 6.3% to 3.4% (*p* < 0.0001). In addition, pivmecillinam prescriptions increased from 47.4% to 52.4% (*p* = 0.042). Kulwicki et al. compared guideline-concordant antibiotic prescribing when an emergency medicine pharmacist was present versus absent, specifically for community-acquired pneumonia or community-acquired intra-abdominal infections [33]. Overall empiric antibiotic prescribing was more likely to be guideline concordant when an emergency medicine pharmacist was present (78% versus 61%, *p* = 0.001). This was consistent across both the community-acquired pneumonia subgroup and the community-acquired intra-abdominal infection subgroup. 

## 4. Discussion

ASP interventions in the ED are diverse, including provider education, prospective culture review, and implementing novel rapid diagnostic methods. The majority of these interventions were able to show a benefit in clinical outcomes or a decrease in antimicrobial use, which suggests that standardization of the treatment of infectious diseases in the ED leads to an improvement in patient care and more appropriate antimicrobial utilization. In addition, the types of infections varied, and some interventions were geared toward stewardship in general or toward certain antimicrobials. Regardless of the method used, all of the interventions led to either improved patient care, decreased antibiotic utilization, or increased guideline concordant therapy. 

Although data exists demonstrating the benefit of stewardship initiatives in the ED, there are still a multitude of disease states where additional opportunities exist for stewardship. Losier et al. conducted a systematic review including 43 studies, which also concluded that antimicrobial stewardship interventions in the ED may improve patient care [34]. The authors focused more specifically on pharmacist involvement in stewardship efforts within the ED, however, they acknowledged that the optimal combination of interventions is still unclear. Further studies are still needed to evaluate the benefit of stewardship initiatives for acute bacterial skin and skin structure infections (ABSSSI), bloodstream infections, central nervous system infections, and intra-abdominal infections. Timbrook et al. concluded that inappropriate antibiotic prescribing was observed in 39% of ED cases in their study [35]. The disease states that had the highest rates observed included bronchitis, upper respiratory tract infections, and skin and soft-tissue infections, and therefore special attention should be given to improving prescribing patterns for these disease states. Similarly, Grenet et al. evaluated the rate of appropriateness of antibiotic prescriptions in a French ED, and approximately 60% did not comply with guidelines [36]. The highest rates of non-compliance were observed in ear, nose and throat infections; skin and soft-tissue infections; respiratory tract infections; and urinary tract infections. 

Although this study highlights multiple interventions that were successfully implemented in the ED, there is a paucity of randomized-controlled trials in the literature. We applaud May et al. and Metlay et al. for their efforts in conducting randomized trials in this area, yet acknowledge that there is a vital need to gain more high-quality data on the treatment of infectious diseases in the ED via randomized studies. In addition to this, although the majority of studies examined in this review led to a positive result, many of the studies sought to examine antibiotic utilization or guideline compliance and did not measure clinical outcomes such as clinical cure, mortality, length of stay, or re-admission rates. More research is necessary to determine whether ASP ED interventions have a significant impact on clinical outcomes in addition to decreasing or optimizing antibiotic use. Additionally, the overwhelmingly positive results available in the literature may be due to publication bias [37]. Finally, although many of the studies reviewed acknowledge the fact that ASP interventions have previously been shown to result in cost savings, the vast majority do not include cost analyses of their own studies. Randomized studies, clinical outcomes data, and cost analyses are all areas of necessity for future research in the area of ASP intervention in the ED.

## 5. Conclusions

Care bundles, guidelines, and antimicrobial stewardship interventions can streamline care and improve the management of common infectious diseases seen in the ED. Since the ED is a fast-paced environment, it is vital that patient care is somewhat standardized, while capitalizing on system- and provider-level interventions while still maintaining provider autonomy. Multidisciplinary involvement that includes physicians, nurses, pharmacists, and mid-level providers has been shown to be beneficial, and is essential to the proper functioning of these bundles and interventions [8]. Pharmacists are a relatively new addition to many ED care teams, and much of the data in this review supports the addition of pharmacists performing culture follow-up after a patient has been discharged from the ED. The ED is the front line for treatment of many infectious diseases, and it is imperative that we begin shifting the focus of our antimicrobial stewardship efforts toward the ED and its entire team. 

## Figures and Tables

**Table 1:** Summary of included studies evaluating antimicrobial stewardship initiatives in the emergency department.

Reference	Study Type	Infection Type	Bundle Elements	Outcomes
	Number of Patients			
**Central Nervous System Infections**				
Viale et al. 2015 [9]	Quasi-experimental study in an Italian hospital ED	Meningitis	-Supportive care -Dexamethasone immediately	In-hospital mortality: 4.7% bundle versus 14.1% control (*p* = 0.04)
	85 patients in bundle group, 92 patients in historical control group		-3^rd^ generation cephalosporin + levofloxacin if turbid CSF	Neurologic sequelae: 13.9% bundle versus 18.9% control (*p* = 0.4)
**Skin and Soft-Tissue Infections**				
May et al. 2015 [10]	Randomized controlled trial in two urban academic EDs	Cutaneous abscesses	-Rapid molecular test implemented to detect *S. aureus* after I&D	Patients with MSSA received β-lactams more often in intervention group (14.5% absolute difference, 95% CI, 1.1% to 30.1%)
	126 patients in intervention group, 126 patients in control group			MRSA positive patients received active antibiotics more often (21.5% absolute difference, 95% CI, 10.1% to 33.0%)
**Respiratory Infections**				
Hortmann et al. 2014 [11]	Retrospective study in German hospital ED	Community-acquired pneumonia	-Education, checklists, institutionalized feedback	In-hospital mortality: 11.3% post-implementation versus 14.3% pre-implementation (*p* = 0.02)
	1325 patients in pre-implementation, 1494 patients in post-implementation			Initiation of antimicrobials within 4 hours: 82.7% post-implementation versus 72.8% pre-implementation (*p* = 0.0001)
				Length of stay: 8 days post-implementation versus 9 days pre-implementation (*p* = 0.02)
Ostrowsky et al. 2013 [12]	Quasi-experimental study at two urban academic EDs	Community-acquired pneumonia	-Development of an algorithm for ED providers, a CAP kit consisting of appropriate antibiotics and dosing regimens bundled with the treatment algorithm, and preloading an automated ED medication dispensing system	Pilot ED appropriate antibiotic selection: 54.9% pre-intervention versus 93.4% post-intervention (*p* = 0.001)
				Second ED appropriate antibiotic selection: 64.6% pre-intervention versus 91.3% post-intervention (*p* = 0.004)
				Antibiotic administration within 6 hours: 85.5% pre-intervention versus 82.1% post-intervention (*p* = 0.48)
Metlay et al. 2007 [13]	Cluster randomized trial at 16 EDs (8 VAs and 8 non-VAs)	Acute respiratory tract infections	-Intervention sites received performance feedback, clinician education, and patient educational materials, including an interactive computer kiosk located in the waiting room	Adjusted antibiotic prescription level for upper respiratory tract infection/acute bronchitis in year 1: 47% for control sites versus 52% for intervention sites
	Control sites: 736 patient visits year one, 736 patient visits year two			Antibiotic prescription change between year one and year two: +0.5% for control sites (95% CI, −3% to 5%) versus −10% at intervention sites (95% CI, −18% to −2%)
	Intervention sites: 840 patient visits year one, 848 patient visits year two			
Dumkow et al. 2018 [14]	Quasi-experimental study at a community teaching ED	Group-A *streptococcus* pharyngitis	-Culture follow-up intervention focusing on symptom assessment and antibiotic avoidance	Antibiotic prescribing at follow-up decreased from 97.0% to 71.3% (*p* < 0.001)
	140 patients in pre-intervention, 140 patients in post-intervention			Appropriateness of therapy at follow-up increased from 6.0% to 81.5% (*p* < 0.001)
				No differences in re-visit at 72 h (*p* = 0.121)
**Urinary Tract Infections**				
Hecker et al. 2014 [15]	Quasi-experimental study in an academic urban ED	Uncomplicated urinary tract infections	-Electronic UTI order set, audit and feedback, financial incentive	Adherence to guidelines: 44% (baseline) to 68% (period one) to 82% (period two) (*p* ≤ 0.015 for each successive period)
	200 patients in pre-intervention, 200 patients in period one post-intervention, 200 patients in period 2 post-intervention			Fluoroquinolone prescriptions: 44% (baseline) to 14% (period one) to 13% (period two) (*p* < 0.001 and p = 0.7 for each successive period)
				Unnecessary antibiotic days of therapy: 250 days to 119 days to 52 days (*p* < 0.001 for each successive period)
Zhang et al. 2017 [16]	Prospective cohort study at a community hospital ED	Asymptomatic bacteriuria	-Pharmacist reviewed all urine cultures and made recommendations to provider	Pharmacist interventions were made for 35/54 (65%) of patients discharged with antibiotics
	136 non-pregnant, asymptomatic patients			Pharmacist interventions for these patients resulted in 122/426 (29%) of potential antibiotic days saved
Hudepohl et al. 2016 [17]	Quasi-experimental study at three Rhode Island EDs	Uncomplicated urinary tract infections	-Education regarding resistance data and preferred antimicrobial therapy	Number of prescriptions: TMP-SMX (13% versus 7%, *p* = 0.01); ciprofloxacin (39% versus 26%, *p* < 0.001)
	1140 patients, 437 prescriptions pre-intervention and 325 prescriptions post-intervention			Ineffective prescriptions: 7.6% pre-intervention versus 4.1% post-intervention (OR 0.51, 95% CI, 0.17 to 1.52)
Percival et al. 2015 [18]	Quasi-experimental study at an academic ED	Uncomplicated urinary tract infections	-Creation of ED specific antibiogram, development of institution-specific antimicrobial recommendations	Choice of therapy consistent with recommendations: 44.8% versus 83% (difference, 38.2%; 95% CI, 33% to 43%; *p* < 0.001)
	174 patients in pre-intervention, 176 patients in post-intervention			Nitrofurantoin use: 12% versus 80% (difference 68%; 95% CI, 62% to 73%; *p* < 0.001)
				Agreement between empiric treatment and the isolated pathogen susceptibility for cystitis: 74% versus 89% (*p* = 0.05)
Landry et al. 2014 [19]	Quasi-experimental study at an academic Canadian ED	Uncomplicated urinary tract infections	-Development and implementation of a best-practice algorithm, physician education	Adherence to best practices: 41% (39/96) pre-intervention versus 66% (50/76) post-intervention (OR 2.81, 95% CI, 1.51 to 5.25, *p* < 0.001)
	96 patients in pre-intervention versus 76 patients in post-intervention			Change in antibiotic selection: OR 0.25, 95% CI, 0.11 to 0.58, *p* < 0.001 driven by a decrease in use of ciprofloxacin, from 32% (31/96) to 11% (8/76)
Jorgensen et al. 2018 [20]	Quasi-experimental study at a community teaching ED	All urinary tract infections	-Development of UTI treatment algorithm emphasizing nitrofurantoin as first line	Increased nitrofurantoin prescriptions (16% to 43%, *p* < 0.001), decreased cephalexin prescriptions (45% to 10%, *p* < 0.001)
	401 patients in pre-intervention, 351 patients in post-intervention		-ASP feedback to providers	Subgroup of those with positive urine culture had fewer return visits if discharged on nitrofurantoin (14% versus 29%, *p* = 0.041)
Rivard et al. 2017 [21]	Quasi-experimental study in an urban ED	*C. trachomatis* and *N. gonorrhoeae* infections	-Initiation of a rapid test for chlamydia and gonorrhea	Increase in treatment appropriateness post-intervention (72.5% versus 60% *p* = 0.008)
	200 patients in post-intervention group, 200 patients in pre-intervention group			Savings of approximately $37,000 per year
**Sepsis**				
Kalich et al. 2016 [22]	Quasi-experimental study at an academic ED	Sepsis—all sources	-Initiation of an antibiotic-specific sepsis bundle, antibiotic dosing recommendations based on source of infection and local susceptibility data, education to providers, antibiotics stocked in automated medication cabinet	Appropriate initial antibiotic: 33.9% versus 54.8% (odds ratio (OR) 0.42, 95% CI, 0.19 to 0.93, *p* = 0.03)
	62 patients in pre-intervention, 62 patients in post-intervention			Appropriate initial antibiotic within 1 h: 22.6% versus 14.5 (OR 1.71, 95% CI, 0.62 to 4.92, *p* = 0.36)
				Appropriate overall antibiotics: 16.1 versus 12.9 (OR 1.30, 95% CI, 0.42 to 4.10, *p* = 0.80)
Viale et al. 2017 [23]	Quasi-experimental study at an Italian ED	Sepsis—all sources	-Sepsis team was created to evaluate the patient within 1 hour and make recommendations for diagnostic work up and therapy	Surviving Sepsis Campaign (SSC) bundle compliance: 4.6% versus 32% (*p* < 0.001)
	195 patients in pre-intervention, 187 patients in post-intervention			Appropriateness of initial antibiotic therapy: 30% versus 79% (*p* < 0.001)
				Predictors of all-cause 14-day mortality: being attended during the post phase was a protective factor (HR 0.64, 95% CI, 0.43 to 0.94, *p* = 0.026)
**Culture Follow-up Programs**				
Santiago et al. 2016 [24]	Single-center, retrospective review study at an academic ED	Positive microbiological results from urine, skin and soft tissue, throat, blood, or stool cultures or other non-culture positive results	-Positive cultures were reviewed by either the EMP or the ED CN for patients discharged from the ED	Median (IQR) time to initial review: 3 (1.0–6.3) hours in EMP group versus 2 (0.3–5.5) hours for the CN group (*p* = 0.35)
	91 cultures in emergency medicine pharmacist group (EMP) versus 87 cultures in charge nurse (ED CN) group			Indicated interventions not completed: 4% (1/25) in EMP group versus 47% (14/30) in CN group (*p* = 0.0004)
Dumkow et al. 2014 [25]	Quasi-experimental study at an academic ED	Urine and blood cultures	-Implementation of a multidisciplinary culture follow-up program in the ED involving pharmacists and ED physicians	Antimicrobial therapy modified in CFU: 25.5% ED re-visits within 72 hours and 30-day readmission: 16.9% in SOC group versus 10.2% in CFU group (*p* = 0.079)
	124 cultures in the standard of care (SOC) group versus 197 cultures in the culture follow-up (CFU) group			Uninsured population ED re-visits within 72 hours: 15.3% in SOC group versus 2.4% in CFU group (*p* = 0.044)
Baker et al. 2012 [26]	Quasi-experimental study at an academic ED	All sources of infection	-Implementation of a pharmacist managed antimicrobial stewardship program. Included education and culture follow-up	Median time to culture review 3 days (range 1–15) in the pre-implementation group versus 2 days (range 0–4) in the post-implementation group (*p* = 0.0001)
	104 cultures in pre-implementation group; 73 cultures in post-implementation group			Median time to patient or PCP notification: 3 days (range 1–9) pre-implementation versus 2 days (range 0–4) post-implementation (*p* = 0.01)
Randolph et al. 2011 [27]	Retrospective study at a single ED	All sources of infection	-Implementation of a pharmacist-run culture follow-up program in the ED	Antimicrobial regimen modifications: 12% in physician managed versus 15% in pharmacist managed
	2278 cultures physician managed versus 2361 cultures pharmacist managed			ED readmission within 96 hours: 19% physician managed versus 7% pharmacist managed (*p* < 0.001)
**Overall Stewardship**				
Dinh et al. 2017 [28]	Quasi-experimental study at a French ED	All sources of infection	-Implementation of an ED antimicrobial stewardship program including a 0.2 FTE ID physician and education	Antimicrobial prescriptions: 769 (3.0%) pre-intervention versus 580 (2.2%) post-intervention (*p* < 0.0001)
	25,470 ED cases pre-intervention versus 26,208 cases post-intervention			Guideline compliance: 285/769 (37%) pre-intervention versus 309/580 (53.3%) post-intervention (*p* < 0.00001)
Kaufman et al. 2017 [29]	Urban community teaching ED	All sources of infection	-Front-line ownership intervention involving ED physicians facilitated by the hospital inpatient ASP	Reduction in antimicrobial use (DDD/1000 ED patient visits): azithromycin −4.573 (*p* = 0.006), ceftriaxone −3.804 (*p* = 0.045), ciprofloxacin −3.340 (*p* = 0.034), and moxifloxacin −9.311 (*p* = 0.008)
	82,617 ED cases in pre-intervention versus 84,980 cases in post-intervention			Rate of urine cultures: decreased by 2.26 urine cultures per 100 ED visits (*p* < 0.001)
Davis et al. 2016 [30]	Retrospective chart review at a single ED	All sources of infection	-Implementation of a pharmacist-driven antimicrobial optimization service	Interventions for inappropriate therapy: 21/42 (50%) nursing managed versus 24/30 (80%) pharmacist managed (*p* = 0.01)
	499 patients with positive cultures in nursing managed period versus 473 patients with positive cultures in pharmacist managed period			Time to intervention: 3.4 ± 1.9 days nursing managed versus 3.5 ± 1.2 days pharmacist managed group (*p* = 0.81)
Borde et al. 2015 [31]	Quasi-experimental study at an academic German ED	All sources of infection, guideline modifications for community-acquired pneumonia	-Guidelines and focused discussion groups emphasize reduced prescription of a third-generation cephalosporin and fluoroquinolones and encourage penicillins	Mean monthly total antibiotic use density: 111 RDD (138 DDD) per 100 patient days pre-intervention versus 86 RDD (128 DDD) per 100 patient days post-intervention
	Antibiotic utilization measured only			Third-generation cephalosporin usage change: −15.2, 95% CI, −24.08 to −6.311
				Aminopenicillin/beta-lactamase inhibitor usage change: +6.6, 95% CI, 4.169 to 9.069
Fagan et al. 2014 [32]	Quasi-experimental study in two Norwegian EDs	Cystitis and pyelonephritis	-Removed ciprofloxacin from the local antibiotic formulary, included a suggestion list for antibiotic use with all point of care urine dipstick testing	Ciprofloxacin prescriptions in intervention ED: 6.3% pre-intervention versus 3.4% post-intervention (*p* < 0.0001)
				Pivmecillinam prescriptions in intervention ED: 47.4% versus 52.4% (*p* = 0.042)
Kulwicki et al. 2019 [33]	Retrospective cohort study in a community teaching ED	Community-acquired pneumonia or community-acquired intra-abdominal infection	-Sought to compare guideline-concordant antibiotic prescribing when an emergency medicine pharmacist (EMP) was present versus absent	Overall empiric antibiotic prescribing was more likely to be guideline-concordant when an EMP was present (78% versus 61%, *p* = 0.001) CAP subgroup (95% versus 79%, *p* = 0.005) CA-IAI subgroup (62% versus 44%, *p* = 0.025)
	185 patients in case group; 135 patients in control group			
